# Identifying Expert Opinions on the Challenges and Barriers Faced in Implementing Iraq’s National Plan for Controlling Hepatitis B

**DOI:** 10.7759/cureus.62814

**Published:** 2024-06-21

**Authors:** Mohammed A Jalal, Manoochehr Karami, Mahshid Namdari, Faris Lami, Taqi Mohammed Jwad Taher, Koorosh Etemad

**Affiliations:** 1 Department of Epidemiology, School of Public Health and Safety, Shahid Beheshti University of Medical Sciences, Tehran, IRN; 2 Department of Family and Community Medicine, College of Medicine in Baghdad University and Al-Sabtain University, Baghdad, IRQ; 3 Department of Family and Community Medicine, College of Medicine, Wasit University, Wasit, IRQ

**Keywords:** barriers, public health, implementation challenges, healthcare professionals, hepatitis b virus

## Abstract

Background: This study examined the difficulties and obstacles faced by healthcare professionals in implementing Iraq's national plan for hepatitis B virus (HBV) control. This research aims to offer valuable insights into the intricacies of HBV control efforts and identify key areas for improvement.

Methods: In this qualitative study, semi-structured interviews were conducted with a purposive sample of 10 physicians, representing diverse medical specialties and healthcare settings, including experts in the fields of medical sciences. Data analysis was conducted using MAXQDA software, version 24 (VERBI Software GmbH, Berlin, Germany) to identify recurring themes and gain insights into the challenges encountered during the implementation of the national plan.

Results: Ten physicians participated in the study, providing insights into challenges and barriers hindering the effective implementation of Iraq's national plan for HBV control. Consensus among participants highlighted challenges such as resource constraints, inadequate infrastructure, population ignorance, and vaccine refusal. Documentation challenges, including inaccuracies in reporting HBV-associated mortality, were also noted. Barriers to successful implementation included poor public awareness, inadequate education for healthcare providers, and funding shortages. Unmet needs highlighted the necessity for unified protocols, surveillance systems, and international training programs. The improvement strategies proposed by participants emphasized raising awareness, supporting primary healthcare centers, and enhancing funding allocation.

Conclusion: This study underscores significant challenges in implementing Iraq's national plan for HBV control, with barriers ranging from resource constraints to communication barriers. Healthcare professionals advocate for targeted interventions, collaborative efforts, and policy measures to address these challenges effectively. The findings contribute to the evidence base for enhancing HBV control efforts in Iraq and emphasize the importance of tailored approaches to public health interventions.

## Introduction

Hepatitis B virus (HBV) infection remains a significant global health challenge, affecting an estimated 257 million individuals worldwide who live with chronic HBV infection. Complications such as cirrhosis and hepatocellular carcinoma lead to approximately 887,000 deaths annually [1-3]. In Iraq, hepatitis B poses a substantial burden on public health, necessitating strategic interventions for control and prevention. To address this challenge, Iraq has implemented a national plan that aims to mitigate the impact of hepatitis B through various prevention and control measures. Iraq's national plan for HBV control focuses on several key strategies: increasing public awareness about hepatitis B, enhancing vaccination coverage, improving and diagnosing HBV infections, and strengthening healthcare infrastructure. The plan also emphasizes the importance of training healthcare providers, ensuring the availability of essential medications and vaccines, and establishing robust surveillance and monitoring systems to track the progress of HBV control efforts [4]. However, the effective implementation of national plans for hepatitis B control is often impeded by numerous challenges and barriers. These obstacles can arise from factors such as limited resources, inadequate infrastructure, socioeconomic disparities, and cultural beliefs that influence healthcare-seeking behaviors [5, 6]. Understanding the intricacies of these challenges is crucial for designing targeted interventions and optimizing the impact of control efforts.

By shedding light on the nuanced challenges faced by healthcare professionals and policymakers, this study provides valuable insights for ongoing efforts to combat HBV in Iraq. Ultimately, the findings of this research have the potential to inform evidence-based strategies and policies tailored to the Iraqi context, thereby advancing the national agenda for HBV control and improving public health outcomes [7].

This study aimed to elucidate the challenges and barriers encountered in the implementation of Iraq's national plan for HBV control by gathering expert perspectives. Through qualitative interviews with experts in fields such as community medicine, epidemiology, and internal medicine, this research seeks to identify key obstacles and opportunities for enhancing the effectiveness of HBV control initiatives in Iraq [8].

This article was previously posted to the Research Square preprint server on May 29, 2024 (https://doi.org/10.21203/rs.3.rs-4428979/v1).

## Materials and methods

Research design

This study utilized a qualitative research design to gain in-depth insights into the challenges and barriers associated with the implementation of Iraq's national plan for HBV control. Qualitative research is well-suited for exploring complex phenomena and understanding the perspectives of key stakeholders. Semi-structured interviews were conducted with experts from various healthcare fields, including community medicine, family medicine, epidemiology, and internal medicine. The interview guide developed specifically for this research project is included as a supplementary file (Appendix A).

Participant selection

Participants were selected based on their expertise and professional experience, specifically targeting physicians from community medicine, family medicine, epidemiology, and internal medicine. The recruitment process involved identifying eligible physicians from various healthcare settings, including hospitals, clinics specializing in hepatitis B treatment, and training primary healthcare centers. A total of 10 participants were chosen to achieve saturation, ensuring no new information or themes would emerge from additional interviews. All participants had a minimum of 10 years of professional experience and were actively engaged in hepatitis B management or related public health initiatives. Eligibility criteria required participants to be licensed physicians in Iraq with at least 10 years of experience in the targeted specialties and familiarity with national healthcare policies and guidelines related to infectious diseases. Physicians who did not meet these criteria, specifically those not familiar with the national guidelines or with less professional experience, were excluded from the study. This rigorous selection process ensured that the participants could provide valuable insights into the challenges and opportunities of implementing Iraq's national plan for HBV control [9].

Data collection

Semi-structured Interviews

The primary method of data collection involved conducting semi-structured interviews with the selected participants. Semi-structured interviews allow flexibility in exploring key topics while also enabling the discovery of emergent themes and responses. The interviews followed a set of open-ended questions designed to cover important areas related to challenges, barriers, opportunities, strengths, and weaknesses associated with the implementation of Iraq's national plan for hepatitis B control.

Informed Consent

Prior to the interviews, participants were provided with informed consent forms that outlined the study's purpose, confidentiality measures, and voluntary participation. Participants were assured of their anonymity and confidentiality throughout the study.

Interview guide development

An interview guide was developed to ensure that key topics were addressed during the interviews. The guide included questions about demographic information, challenges encountered in implementing the national plan, barriers to effective control measures, opportunities for improvement, and the strengths and weaknesses of existing strategies.

Data analysis

The analysis of qualitative data was carried out using MAXQDA software version 24 (VERBI Software GmbH, Berlin, Germany), a comprehensive tool designed for organizing, coding, and analyzing interview data. The analysis process commenced by iteratively coding the interview transcripts, and systematically assigning labels to segments of text to identify recurring themes, sub-themes, patterns, and insights pertaining to the challenges encountered during implementation. Through a process of constant comparison and refinement, themes were developed to encapsulate the essence of participants' experiences and perspectives.

Sample saturation

Sample saturation, a key aspect of qualitative research, was achieved when no new themes or insights emerged from additional interviews, indicating that the data collection had reached a point of redundancy. This was confirmed through a systematic review of interview transcripts, where all opinion comments were found to be similar, suggesting a comprehensive exploration of the research topic [10].

## Results

Ten physicians from diverse medical specialties, including epidemiology, family medicine, internal medicine, and community medicine, were interviewed for this study. The participants had 10 to 40 years of experience and were affiliated with various healthcare settings, such as public health departments, medical colleges, clinics, and hospitals. Their involvement in healthcare programs varied, with some directly engaged in public health initiatives or community outreach programs.

Despite their diverse backgrounds, all participants shared a common goal of addressing the challenges associated with implementing Iraq's national plan for HBV control, as indicated in Table 1. While most participants reported active engagement in healthcare programs, their levels of training on hepatitis B varied. Some participants had received specific training on hepatitis B, while others had not. Interestingly, none of the participants were currently involved in HBV research or publications, although several were actively involved in public health initiatives or community outreach efforts. Furthermore, the majority of participants demonstrated familiarity with national healthcare policies and guidelines related to infectious diseases in Iraq, indicating their awareness of the regulatory frameworks and standards governing healthcare practices in the country.

**Table 1:** Summary of participant characteristics and involvement in hepatitis B virus management and public health initiatives in Iraq (n = 10) Hosp: hospital; MC: medical college; PHD: public health department; IM: internal medicine; FM: family medicine; CM: community medicine; Epid: epidemiologist

Years of experience	Healthcare setting	Medical specialty	Involvement in healthcare programs	Training on hepatitis B	Involvement in hepatitis B research/publications	Involvement in public health initiatives/community outreach	Familiar with national healthcare policies/guidelines
15	Hosp	IM	Yes	Yes	No	No	Yes
17	Hosp	IM	No	Yes	No	No	No
32	MC	FM	Yes	Yes	No	Yes	Yes
32	PHD	IM	Yes	Yes	No	Yes	Yes
35	MC	CM	Yes	No	Yes	Yes	Yes
17	PHD	Epid	Yes	Yes	No	Yes	Yes
10	Clinic	FM	No	No	No	Yes	Yes
31	PHD	Epid	Yes	Yes	No	Yes	Yes
10	PHD	Epid	Yes	Yes	No	No	Yes
40	PHD	Epid	Yes	No	No	Yes	Yes

Based on expert opinions and precise insights, a comprehensive analysis was conducted to examine the implementation of the HBV control strategy in Iraq. This analysis systematically explores various themes and sub-themes, providing a detailed understanding of the challenges that impede effective execution. These challenges include resource constraints, documentation deficiencies, inadequate public awareness, and funding shortages. However, the analysis also identified opportunities for improvement, such as enhancing health education and strengthening healthcare infrastructure. By shedding light on the complexities involved in HBV control efforts, this analysis not only serves as a roadmap for addressing critical gaps but also contributes to progress in Iraq's public health landscape as demonstrated in Table 2.

**Table 2:** Themes and subthemes identified in the study on implementing Iraq's national plan for HBV control GIT: gastrointestinal tract; HBV: hepatitis B virus

No.	Themes and subthemes	Codes
1	Challenges	
	Implementation challenges	Resource constraints and limited infrastructure; population ignorance and vaccine refusal; financial issues and difficult communication with patients; inaccurate data and incomplete patient addresses
	Documentation challenges	Registration error of the cause of death-on-death certificates; instances where patients pass away at home without being officially registered; insufficient orientation of medical students regarding the risks of hepatitis B; limited access to standardized reporting tools and systems.; fragmented electronic systems; tendency to report most deaths as a hepatic failure without specifying the underlying hepatitis
2	Barriers	
	Barriers to implementation	Poor public awareness and response; insufficient education for healthcare providers and the general population; inadequate funding and a shortage of trained personnel; inadequate documentation practices; lack of media support and health promotion programs
	Unmet needs	Availability of unified protocols or guidelines; surveillance and monitoring of laboratories and investigations; international training to enhance healthcare professionals' experience and knowledge; ensuring availability of vaccines; adequate resources for comprehensive patient education and access to affordable treatment options; implementing mandatory vaccination policies; regulating or controlling practices such as cupping, tattooing, and piercing; addressing inadequacies of laboratory tests
3	Opportunities	
	Improvement strategies	Raising awareness about health issues and imposing penalties on violators; supporting primary health centers, community doctors, GIT centers, blood banks, vaccination programs, and infection control units; reinstating previous plans and staff; providing training opportunities for untrained personnel; boosting financial support for medical institutes.; ensuring the availability of necessary medication and vaccines; educating patients about various diseases; establishing medical centers within each Department of Health (DOH)
4	Weaknesses and strengths	
	Weaknesses and areas for improvement	Lack of serial screening for healthcare providers and other high-risk groups; inadequate availability of drugs for treatment; weak training of healthcare providers on sterilization practices and post-needle stick actions; absence of mandatory requirements for individuals to adopt prevention strategies; instances of fraudulent or inaccurate results in premarital investigations; inadequately established and consistent protocols for both private and governmental healthcare settings; insufficient recording and reporting systems; discontinuity of services; insufficient public awareness campaigns about hepatitis B; limited access to treatment options; decreased patient awareness about the disease; low vaccination rates among healthcare workers; inadequate support for healthcare workers
	Strengths and positive experiences	Effective interventions for addressing hepatitis B infection; successful implementation of Iraq's national immunization schedule for children under five
5	Mortality and treatment related to HBV	Challenges in accurately documenting and reporting mortality related to HBV; strategies or interventions found effective in ensuring the effective management and treatment of patients with HBV

Challenges

Implementation Challenges

The main challenges that have been encountered in implementing Iraq’s national plan for HBV control in medical practice were discussed. When asked about the main challenges, seven physicians identified resource constraints, limited resources and infrastructure, population ignorance, lack of international training and stakeholder experience, vaccine refusal, financial issues, difficult communication with patients, and inaccurate data as significant obstacles. Additionally, two other physicians emphasized challenges such as incorrect or incomplete patient addresses, a lack of community awareness, the spread of cupping and tattooing practices, insufficient commitment by health institutions to preventive measures, and fluctuations in examination materials and medications. To address these challenges, it is crucial to promote teamwork and prioritize sanitation and preventive measures. Other necessary actions include increasing vaccination rates, conducting regular surveys for hepatitis B virus among food handlers, and providing orientation for healthcare workers.

Documentation Challenges

The challenges that have been encountered in accurately documenting and reporting mortality related to the hepatitis B virus within medical practice.

Three physicians identified several challenges related to accurately documenting and reporting mortality associated with hepatitis B. These challenges included the misregistration of the cause of death on death certificates, instances where patients pass away at home without being officially registered, insufficient orientation of medical students regarding the risks of hepatitis B, limited access to standardized reporting tools and systems, fragmented electronic systems, financial issues, decreased healthcare provider knowledge regarding technology, and a tendency to report most deaths as hepatic failure without specifying the underlying hepatitis.

Additionally, six physicians highlighted the difficulty of attributing deaths solely to HBV due to the chronic nature of the disease, its complications, and the involvement of other organs.

Barriers

Barriers to Implementation

In the participants’ opinions, the key barriers to the successful implementation of the national plan in the context of medical practice are listed below.

Five physicians identified several key barriers to the successful implementation of the national plan. These barriers included poor public awareness and response, insufficient education for healthcare providers and the general population, inadequate funding and a shortage of trained personnel, inadequate documentation practices, and a lack of media support and health promotion programs. Furthermore, four physicians emphasized the need for advanced development in primary healthcare centers to ensure effective treatment, vaccination programs, and health education. They also highlighted the barrier posed by the lack of experience and training among managers.

Unmet Needs

This section documents the specific needs or requirements that have not been adequately addressed in the context of managing and controlling HBV within the healthcare setting.

All 10 physicians unanimously agreed on the unmet needs for managing and controlling the hepatitis B virus. These needs include the availability of unified protocols or guidelines encompassing all types of medical care. They emphasized the importance of the surveillance and monitoring of laboratories and investigations to ensure accurate and reliable results. The physicians also emphasized the need for international training to enhance healthcare professionals' experience and knowledge in managing hepatitis B.

Additionally, they highlighted the necessity of ensuring the availability of vaccines to effectively prevent the spread of the virus. Adequate resources for comprehensive patient education and access to affordable treatment options were also identified as crucial needs. Furthermore, the physicians stressed the importance of implementing mandatory vaccination policies as well as regulating or controlling practices such as cupping, tattooing, and piercing, which may pose a risk for hepatitis B transmission. Finally, addressing the inadequacies of laboratory tests was highlighted as an essential aspect of managing and controlling the virus effectively.

Opportunities

Improvement Strategies

The improvements or additional support that enhance the implementation of Iraq’s national plan for HBV control in the healthcare setting were discussed.

All 10 physicians unanimously agreed on several improvement strategies for the implementation of Iraq's national health plan for HBV control. These strategies include raising awareness about health issues and imposing penalties on violators. They emphasized the importance of supporting primary health centers, community doctors, gastrointestinal tract (GIT) centers, blood banks, vaccination programs, and infection control units. To enhance primary healthcare services, they recommended the reinstatement of previous plans and staff, providing training opportunities for untrained personnel, and giving more attention to junior doctors.

Furthermore, the physicians stressed the need for additional support and resources, such as comprehensive training programs and increased funding allocation. They emphasized the importance of boosting financial support for medical institutes, ensuring the availability of necessary medication and vaccines, providing healthcare provider training, and educating patients about various diseases. They also suggested that laws and legislation mandate full vaccination for all high-risk groups. Additionally, it was recommended to establish a medical center within each Department of Health (DOH) to address the comprehensive medical needs of patients.

Weaknesses and strengths

Weaknesses and Areas For Improvement 

The participants’ opinions, weaknesses, or areas requiring improvement in the current implementation of Iraq’s National Plan for Hepatitis B Virus Control within medical practice were noted.

Five physicians identified several weaknesses and areas for improvement in the current implementation of healthcare for hepatitis B. These include the lack of serial screening for healthcare providers and other high-risk groups, inadequate availability of drugs for treatment, weak training of healthcare providers on sterilization practices and post-needle stick actions, the absence of mandatory requirements for individuals to adopt prevention strategies, instances of fraudulent or inaccurate results in premarital investigations, insufficiently established and consistent protocols for both private and governmental healthcare settings, inadequate recording and reporting systems, discontinuity of services, insufficient public awareness campaigns about hepatitis B, limited access to treatment options, decreased patient awareness about the disease, low vaccination rates among healthcare workers, and inadequate support for healthcare workers. Addressing these weaknesses and areas for improvement is crucial to enhancing the overall provision of healthcare for patients with hepatitis B and ensuring better outcomes.

Strengths and Positive Experiences

Strengths or successful experiences were observed in the context of implementing Iraq’s national plan for HBV control in medical practice.

Four physicians highlighted several effective interventions for addressing hepatitis B infection. These interventions included promoting good personal hygiene practices, implementing effective vaccination and screening programs, providing training and education for healthcare providers, ensuring proper sterilization protocols for health authorities, offering premarital health services, establishing robust recording and reporting systems, making vaccines readily available, implementing early detection initiatives, conducting patient education programs, and promoting health education and awareness. These interventions have demonstrated positive outcomes in controlling the spread of the hepatitis B virus.

Three physicians acknowledged the successful implementation of Iraq's national immunization schedule for children under five. They noted that the hepatitis B vaccine is readily available free of charge at primary healthcare centers. The Ministry of Health (MOH) has provided support for this initiative, resulting in increased awareness among healthcare professionals and patients regarding disease and preventive measures. Additionally, medical care related to hepatitis B is provided free of charge. The presence of a clear action plan and the availability of vaccines have facilitated the screening of high-risk groups.

Mortality and treatment related to HBV

The text passages discuss mortality and treatment related to HBV. This highlights that physicians sometimes fail to register the cause of liver cirrhosis or liver failure in death certificates, resulting in a lack of research data. To improve data collection, electronic registration, and standardized reporting protocols are recommended. Access to affordable medication and specialized care is crucial for effective treatment. Prevention measures, such as vaccination, controlling sources of infection, and implementing effective screening in blood banks, are emphasized. Patient education and awareness play a vital role in improving treatment outcomes and reducing complications. Overall, enhancing healthcare providers’ knowledge, technology, and training is necessary for effective HBV management.

Questions that arose during a detailed discussion on the topic

Question 1: How do you perceive the current level of access to treatment for hepatitis B virus patients in your healthcare setting, and what improvements do you think are necessary in this area?

Answer: "The current level of access to treatment for hepatitis B virus patients in our healthcare setting is inadequate. There are challenges in ensuring a consistent supply of medication to healthcare institutes, and the availability of specialized care is limited. Improvements are necessary in terms of making medication more affordable and accessible to patients. Additionally, there is a need for better availability of laboratory investigations and medication in healthcare settings."

Question 2: In your experience, what measures or systems do you believe would facilitate the accurate registration and documentation of mortality related to HBV within the healthcare system?

Answer: "To facilitate accurate registration and documentation of mortality related to HBV within the healthcare system, several measures and systems can be implemented. First, the adoption of electronic registration and standardized electronic database systems would streamline the process and ensure accurate data collection. Capacity building within health systems is also crucial for enhancing the knowledge and skills of healthcare providers in accurately documenting and reporting mortality cases. Standardized reporting protocols should be established to ensure consistent and comprehensive reporting. The use of electronic medical records can further improve the accuracy and efficiency of documentation. Additionally, providing training for accurate completion of death certificates and utilizing recent technology for documentation would contribute to better registration and documentation practices. Central surveillance and monitoring of guidelines and protocols by the MOH are also necessary to ensure accurate reporting."

Question 3: What challenges have you encountered in accurately documenting and reporting mortality related to the hepatitis B virus within your medical practice?

Answer: "In our medical practice, we have encountered several challenges in accurately documenting and reporting mortality related to the hepatitis B virus. One challenge is that physicians often fail to register the specific cause of liver disease on death certificates, which leads to incomplete documentation. Inconsistent reporting standards and a lack of integration with national databases also pose challenges to obtaining accurate data. Financial constraints may affect healthcare providers' access to technology and training, which can impact the accuracy of documentation. Additionally, some patients may pass away before receiving a diagnosis, making it difficult to document the cause of death accurately."

Question 4: What strategies or interventions have you found effective in ensuring the effective management and treatment of patients with the hepatitis B virus in your clinical practice?

Answer: "In our clinical practice, we have found several strategies and interventions effective in ensuring the effective management and treatment of patients with the hepatitis B virus. The implementation of an efficient vaccination program has been crucial for preventing new infections. Controlling sources of infection, especially in illegal places, has also been effective in reducing the spread of HBV. Utilizing recent and advanced laboratory techniques for screening in blood banks helps identify infected individuals and prevent transmission through blood transfusions. Providing effective management after accidental needlestick incidents is essential to protecting healthcare workers. Regular monitoring, early intervention, and patient education programs have been successful in managing the disease and preventing complications. Patient education about the disease and the availability of antiviral medication are important components of effective management and treatment."

## Discussion

Hepatitis B virus (HBV) infection presents a significant global public health challenge, and Iraq is no exception to its impact. This study provides insights into the challenges and barriers faced in implementing Iraq's national plan for HBV control from the perspective of healthcare professionals. Through qualitative interviews with experts from various medical specialties, this study offers valuable insights into the complexities of HBV control efforts and identifies areas for improvement.

Challenges in implementation

This study reveals several challenges that hinder the effective implementation of Iraq's national plan for HBV control. These challenges include limited resources, inadequate infrastructure, population ignorance, and cultural beliefs influencing healthcare-seeking behaviors. Additionally, issues such as vaccine refusal, financial constraints, communication difficulties with patients, and inaccurate data pose significant obstacles to control efforts [11-13, 2].

Documentation challenges

Documentation challenges related to accurately reporting HBV-associated mortality highlight the limitations of current healthcare systems in capturing precise data. Issues such as misregistration of causes of death, unregistered deaths, and inadequate surveillance systems necessitate investments in healthcare infrastructure, training programs, and digital health solutions to enhance data collection and analysis [14-18].

Barriers to successful implementation

This study identifies barriers to successful implementation that align with common challenges observed in healthcare systems in resource-limited settings. These barriers include poor public awareness and response, inadequate education for healthcare providers and the general population, and funding shortages. Furthermore, insufficient media support and health promotion programs exacerbate this situation, emphasizing the need for multisectoral collaboration and effective communication strategies [19-22].

Unmet needs

This study identified unmet needs in HBV control efforts, including the need for unified protocols, surveillance systems, and international training programs. Additionally, there is a focus on vaccine availability, resources for patient education, and regulatory frameworks to address both supply-side and demand-side factors influencing healthcare delivery [23, 24].

Improvement strategies

Participants in the study proposed improvement strategies that emphasize raising awareness, supporting primary healthcare centers, and increasing funding allocation for HBV control efforts. These strategies align with global health frameworks such as sustainable development goals and stress the importance of policy interventions, mandatory vaccination policies, and the regulation of risky practices [25-27].

Implications for policy and practice

The findings underscore the need for targeted interventions that address the specific challenges identified by healthcare professionals. Policymakers are urged to prioritize investments in healthcare workforce development, capacity building, and collaborative efforts involving government agencies, nongovernmental organizations, and community stakeholders to implement comprehensive HBV control strategies [22, 28, 29].

## Conclusions

A study on Iraq's national plan for HBV control revealed significant challenges in its implementation, including resource constraints, communication barriers, and documentation issues. Healthcare professionals identify barriers such as poor public awareness, funding shortages, and inadequate education. Improvement strategies focus on raising awareness, supporting healthcare centers, and enhancing funding allocation. Unmet needs highlight the importance of unified protocols, surveillance systems, and international training. This study underscores the necessity for targeted interventions, collaborative efforts, and policy measures to enhance HBV control in Iraq and effectively address the identified challenges.

## Appendices

Appendix A

Interview Guide

5-    Semi-structure of the interview:                             No:

Domain One: Demographic Information

1.     Personal Information:

a.     Full Name: _______ (To add names as advisors in developing the national plan in the future if necessary)

b.     Email: _______ (To benefit from important suggestions if necessary)

c.      Gender: Male / Female _______

2.     Professional Background:

a.      Medical Specialty: _______

b.     Years of Experience in Healthcare: _______

c.      Healthcare Setting: Hospital / Clinic /Public Health Department/ Health Care Center / Other (please specify): _______

d.     Are you directly involved in the management or implementation of healthcare programs? Yes / No

e.     Have you received specific training related to Hepatitis B virus management and control? Yes / No

f.      Are you currently involved in research or publications related to the Hepatitis B virus? Yes / No

g.     Are you currently involved in any public health initiatives or community outreach programs? Yes / No

h.     Are you familiar with the current national healthcare policies and guidelines related to infectious diseases in Iraq? Yes / No

A.    Domain Two: Challenges

1.     Implementation Challenges: What are the main challenges you have encountered in implementing Iraq’s National Plan for Hepatitis B Virus Control in your medical practice? _______

2.      Documentation Challenges: What challenges have you encountered in accurately documenting and reporting mortality related to Hepatitis B virus within your medical practice? _______

3.     Resource Support: How do you perceive the level of support and resources provided for the implementation of the national plan in your healthcare setting? _______

B.    Domain Three: Barriers

1.     Barriers to Implementation: In your opinion, what are the key barriers to a successful implementation of the national plan in the context of your medical practice? _______

2.     Unmet Needs: What specific needs or requirements have not been adequately addressed in the context of managing and controlling Hepatitis B virus within your healthcare setting? _______

C.    Domain Four: Opportunities

1.     Improvement Strategies: What improvements or additional support do you think would enhance the implementation of Iraq’s National Plan for Hepatitis B Virus Control in your healthcare setting? _______

2.     Effective Strategies: What strategies or interventions do you believe are most effective in addressing Hepatitis B virus control within your medical specialty? _______

D.    Domain Five: Weaknesses and Strengths

1.     Areas for Improvement: In your opinion, what are the weaknesses or areas requiring improvement in the current implementation of Iraq’s National Plan for Hepatitis B Virus Control within your medical practice? _______

2.     Positive Experiences: What strengths or successful experiences have you observed in the context of implementing Iraq’s National Plan for Hepatitis B Virus Control in your medical practice? _______

3.     Effective Interventions: In your experience, what interventions or practices have shown positive outcomes in addressing Hepatitis B virus control within your healthcare setting? _______

E.    Domain Six: Mortality and Treatment Related to HBV:

1.     What challenges have you encountered in accurately documenting and reporting mortality related to Hepatitis B virus within your medical practice? _______

2.     What strategies or interventions have you found effective in ensuring the effective management and treatment of patients with Hepatitis B virus in your clinical practice? _______
